# Possible Demonstration of a Polaronic Bose-Einstein(-Mott) Condensate in UO_2(+x)_ by Ultrafast THz Spectroscopy and Microwave Dissipation

**DOI:** 10.1038/srep15278

**Published:** 2015-10-16

**Authors:** Steven D. Conradson, Steven M. Gilbertson, Stephanie L. Daifuku, Jeffrey A. Kehl, Tomasz Durakiewicz, David A. Andersson, Alan R. Bishop, Darrin D. Byler, Pablo Maldonado, Peter M. Oppeneer, James A. Valdez, Michael L. Neidig, George Rodriguez

**Affiliations:** 1 Synchrotron Soleil, L’Orme des Merisiers Saint-Aubin, BP 48 91192, Gif-sur-Yvette, France; 2 Los Alamos National Laboratory, P.O. Box 1663, Los Alamos, NM 87545, USA; 3 Department of Chemistry, University of Rochester, NY 14627, USA; 4 Department of Physics and Astronomy, Uppsala University, S-75120, Uppsala, Sweden

## Abstract

Bose-Einstein condensates (BECs) composed of polarons would be an advance because they would combine coherently charge, spin, and a crystal lattice. Following our earlier report of unique structural and spectroscopic properties, we now identify potentially definitive evidence for polaronic BECs in photo- and chemically doped UO_2(+x)_ on the basis of exceptional coherence in the ultrafast time dependent terahertz absorption and microwave spectroscopy results that show collective behavior including dissipation patterns whose precedents are condensate vortex and defect disorder and condensate excitations. That some of these signatures of coherence in an atom-based system extend to ambient temperature suggests a novel mechanism that could be a synchronized, dynamical, disproportionation excitation, possibly via the solid state analog of a Feshbach resonance that promotes the coherence. Such a mechanism would demonstrate that the use of ultra-low temperatures to establish the BEC energy distribution is a convenience rather than a necessity, with the actual requirement for the particles being in the same state that is not necessarily the ground state attainable by other means. A macroscopic quantum object created by chemical doping that can persist to ambient temperature and resides in a bulk solid would be revolutionary in a number of scientific and technological fields.

Because of their formation from the ground states of simple or composite bosons, atomic Bose-Einstein condensates^1^,^2^,^3^ were and remain a distinctly ultralow temperature phenomenon that turns on at low densities where the distance between the constituent particles in their ground states approaches their thermal wavelength. The low masses and higher densities of certain quasiparticles in solids– magnons^4^,^5^,^6^, polaritons^7^,^8^, and excitons^9^,^10^,^11^,^12^ – have allowed their condensates to be extended to room temperature under certain conditions^5^,^13^,^14^. These findings, and the accuracy of associated theories, suggest that condensation is neither particularly complicated nor special. This poses the question of whether more exotic types of condensates could exist, perhaps made of more exotic, i.e., strongly interacting, types of quasiparticles. An especially interesting candidate could be polarons because of their coupling of charge, spin, and lattice degrees of freedom as well as their connection to exotic superconductivity. The polaronic Bardeen-Cooper-Schrieffer (BCS) condensate in high temperature superconductors may, in fact, be complemented by BECs^15^ that are a likely component of two-band superconductivity^16^,^17^,^18^,^19^,^20^,^21^.

We have previously described structural and both ultrafast optical pump-probe and static x-ray absorption spectroscopy experiments on the chemically and photo-doped, partly filled 5*f* Mott insulator UO_2(+x)_ that we interpreted as the signatures of superfluid droplets of aggregated polarons that self-organized as coherent bosonic states^22^. We now report: 1) optical pump-terahertz time domain spectroscopy (TTDS) measurements that display the coherence that is a defining characteristic of a condensate; and 2) electron paramagnetic resonance (EPR), i.e., microwave absorption spectra, that exhibit dissipation and some very broad, complicated, highly temperature dependent features. This dissipation is like that in cuprate superconductors^23^,^24^ where it was attributed to vortex reordering,^25^,^26^,^27^ a phenomenon that also occurs with BECs^28^,^29^,^30^, and the features are similar to the distinct excitations also found in BECs but at radiofrequency energies consistent with their much lower stability^31^,^32^,^33^,^34^,^35^. These mesoscale condensates would be composed of composite bosons formed from phase-coherent fermionic polarons that are charge defects in the lattice and not quantum fluctuations^35^,^36^. The formation of these bosons by a dynamic charge transfer between the U ions and their condensation and display of superfluid properties are promoted by a Feshbach resonance, analogous to the formation of the Cooper pairs that give the pseudogap phase and its subsequent condensation in ultracold fermionic atom gases promoted by this type of resonance facilitating the conversion between the original atoms and bosonic diatomic molecules^37^. The multiple quantum phases we detect are indicative of the spatial and temporal variations of these coherent domains and their strong interactions with the lattice. Of particular interest is that the properties signifying the condensate persist through ambient temperature despite the large masses involved, indicating a novel BEC formation mechanism. The experimental evidence indicates a dynamic mechanism instead of the conventional static one, which would be a collective charge-transfer disproportionation excitation originating in the confluence of the valence fluctuations of the dynamical polarons and the specific structural chemistry of the UO_2+x_ system^22^. This is particularly interesting because it would demonstrate that macroscopic coherence can be obtained not only by placing the constituent particles in their ground state but also more generally by other processes that synchronize them temporally and spatially.

Partly filled Mott insulators exhibit a variety of fascinating properties. In actinide dioxides in particular, the 5*f* character of excitations across the Mott gap may cause other free electron “in gap” states (IGS)^38^,^39^ in proximity to the Fermi level^40^. These would be in addition to the polaronic states within the gap from chemical doping^41^. The interactions or even combination of these two types of IGS could be the origin of the unusual structural chemistry of uranium oxides with valences ≥IV, much as it is in two-band models of high temperature superconductors^42^,^43^. In particular, the binary^44^ and ternary^45^,^46^,^47^,^48^ uranium oxides exhibit an essentially continuous range of U-O bond lengths as their geometries range from highly oblate to perfectly octahedral, defying the normal coupling of valence to geometry. The pair potential controlling the U-O distances must therefore be relatively flat, causing the radial ground and oblate excited state symmetries to intersect more as a conic section than the normal cusp. This property promotes the interchange between the two states and renders dynamics an important component of the structure and other properties^49^. The valence-structure dynamics and subsequent non-adiabatic-non-Born-Oppenheimer spin-charge-lattice fluctuations of the anharmonic, double well potential of the polarons^50^ in cuprates^51^,^52^,^53^,^54^, manganites^55^,^56^, and other correlated oxides manifest as differences in the structures found by the different time domains of neutrons vs. x-rays^57^,^58^. These properties are greatly magnified in UO_2+x_^22^, which also exhibits the predicted broadening^57^ for the 5*f*-6*d* electron manifold. By influencing the dynamics of excited states^40^,^59^ these polarons evolve into exceptional quasiparticles whose interactions could form a variety of bosonic quantum phases.

## Results and Discussion

**Figure 1:**
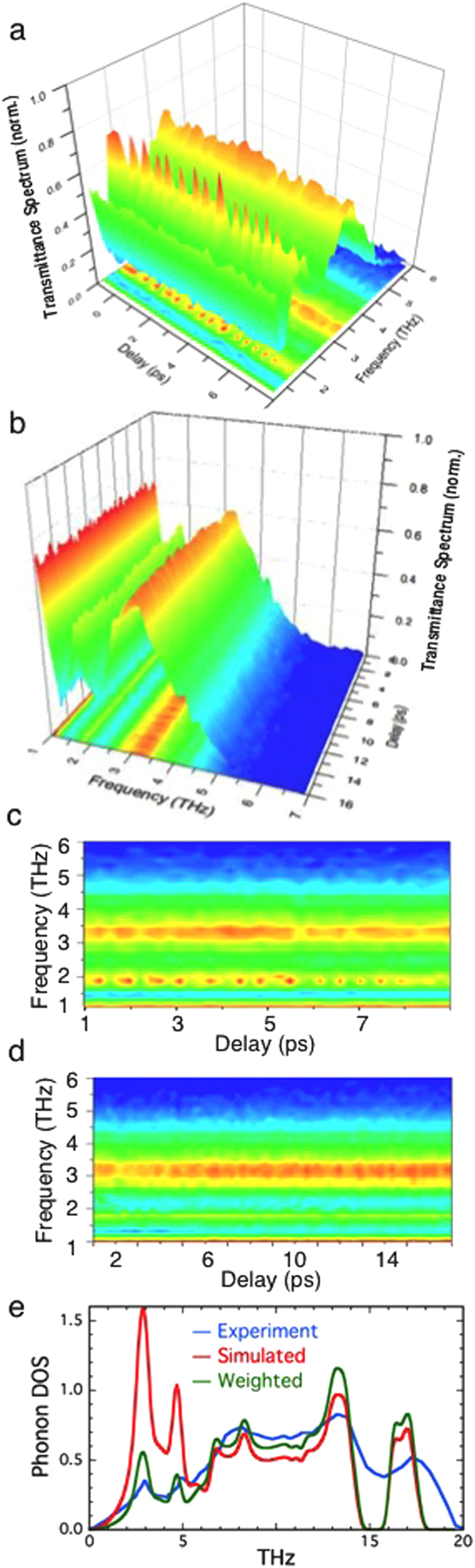
Time evolution of optical pump-TTDS probe experiments. **(a)** Frequency of the normalized transmission as a function of time up to 8 ps following excitation at 1.57 eV. Regular modulation of the amplitude on top of the peak at 1.8 THz is apparent. **(b)** Frequency as a function of time following excitation at 3.14 eV, across the Mott gap, which does not show oscillations. **(c/d)** Overhead views of (a/b), with the colors representing the same amplitude of the normalized transmittance. (**e**) Calculated and experimental phonon DOS. The dispersion curves giving the DOS are in Supplementary Fig. S1 online.

*Optical pump-TTDS probe experiments on UO*_*2*_. The defining property of a condensate is its coherence, spatially over dozens to hundreds of μm and temporally, in conventional BECs, msec. This is manifested statically as evidence that the 10^3^–10^6^ constituent particles are synchronized by, e.g., exhibiting position and momentum distributions much narrower than the expected thermal ones^1^,^2^,^3^,^5^,^7^,^12^. The coherence of the constituent particles of condensates frequently manifests itself as oscillatory or periodic behavior and interference patterns, especially in their excitations^14^,^60^,^61^,^62^,^63^,^64^,^65^,^66^. We show here the Fourier-transformed TTDS that contains the combined momentum-averaged phonon density of states and, particularly at low energies, electronic or other excitations that in UO_2.00_ would be the IGS and/or collective states. On pumping at both 3.14 eV (5*f*→5*f*) and 1.57 eV (5*f*→IGS) the THz spectra display a peak around 1.8 THz (Fig. 1a–d, precision limited by the sampling interval dictated by the sample thickness) that is not a UO_2_ phonon (Fig. 1e)^67^,^68^. Although emission was not measured, the sensitivity of the absorption to the relative orientation of the crystal, the pump, and the probe beams was quite high, indicating a strong correlation between the lattice vectors and the excited state over the duration of the measurement. The experimental geometry and the fact that the 1.8 THz feature is lower in amplitude than the other features in this transmission measurement demonstrate that it originates in absorption and not emission.

**Figure 2:**
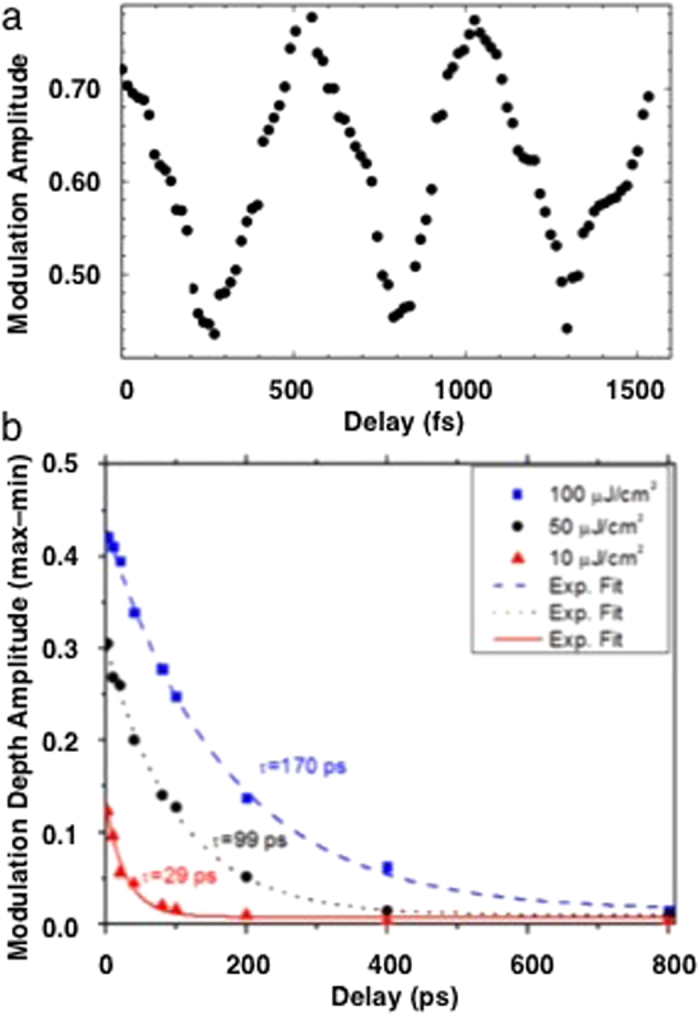
(a) Time dependence of the amplitude of the 1.8 THz peak showing the structure of the oscillations. **(b)** Time dependence–exponential decay of the oscillation amplitude depth at three different fluences.

The observation of the 1.8 THz state and its extended lifetime after inducing the metal-to-metal-charge-transfer transition with 3.14 eV excitation into the primarily 5*f* portion of the upper Hubbard band (Fig. 1B) are both exceptional. However, pumping at 1.57 eV directly into the gap most remarkably also gives the distinct, regular oscillations in the amplitude of this 1.8 THz peak throughout its two-hundred picosecond lifetime (Figs 1a, c and 2). These are a direct demonstration of spatial and temporal coherence in the corresponding state or states throughout the probed volume. The oscillations correspond to fluctuations in the density of the free electrons in the IGS that are the origin of this spectral feature. This modulation therefore corresponds to a phase coherent modulation in the quantity of the polaronic liquid phase, either by direct exchange with a second state that does not give a signal in these spectra or via relaxation and subsequent restoration of these electrons to their original U 5*f* state. The excitation dependence indicates that coherence within the 1.8 THz state is not coupled to the direct transition to the upper Hubbard band but is instead correlated with free electrons within the IGS ~8 meV above the chemical potential. The coherence is not only spatial via some type of long range interactions, it is also maintained temporally, with its amplitude still ≥1/e of its initial value after 170 ps and hundreds of oscillations (Fig. 2B). This can be compared with the few to a few dozen oscillations observed with normal fermionic condensates and gases^37^,^64^. Even without the oscillations, the extended lifetime of the photoinduced polarons that give this spectral feature is likely be a consequence of the coherence as well. In addition, this potential stability provided by the condensate and the possibility of spontaneous or facilitated population of the IGS offers explanations for the EPR signals reported below, the photoinduced polarons produced here by pumping into the gap, and other results obtained with UO_2.00_ such as the U-oxo species found by EXAFS in ion-irradiated neutral UO_2_. An important point is that since the TTDS is a transmission experiment the collective excitations of the individual or aggregated polarons that give the oscillations must be synchronous throughout the entire approximately 0.1 mm^3^ volume of the sample that is illuminated and probed so that the coherence is maintained within a quantity of material that can be weighed on a standard balance.

**Figure 3:**
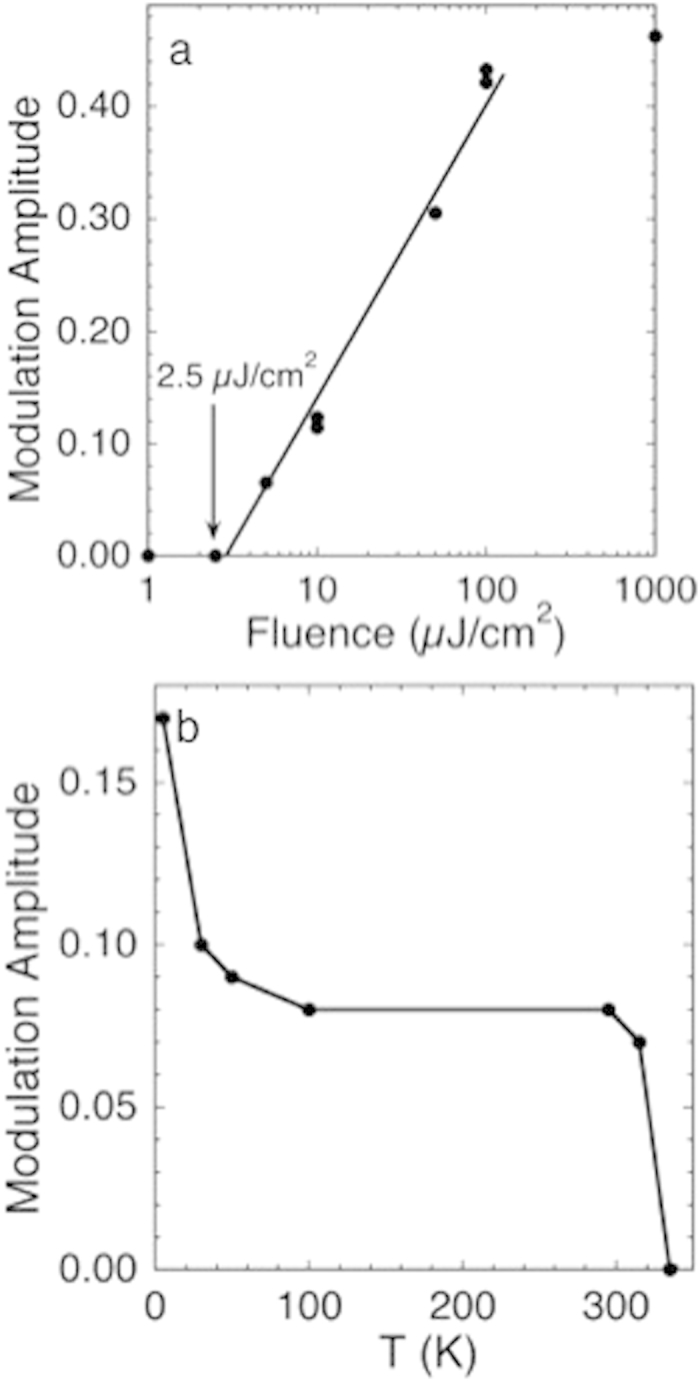
(a) Fluence dependence of the amplitude of the 1.8 THz peak at its inception. The arrow points to the onset of oscillations, the line is a guide through the region where the amplitude is increasing linearly. Duplicate measurements were performed at 10 and 100 μJ/cm^2^. **(b)** The initial oscillation amplitude as a function of temperature.

As the fluence is increased the amplitude of the 1.8 THz oscillation shows an initial threshold, a linear region, and saturation (Fig. 3a). This would be expected from a coherent polaronic superfluid that requires a minimum quasiparticle concentration for aggregation and self-organization into the domains of the polaronic liquid phase but also will have a limiting density derived from the optimum overlap of the bosonic wavefunctions. Since the constituent quasiparticles are transient there will also be a contribution from their lifetimes that are extended in the coherent state. Thus the increase in oscillation amplitude below 100 K (Fig. 3b) can be attributed to reduced quasiparticle scattering. At higher temperatures the oscillation amplitude is the same at 100 and 295 K and then diminishes to zero at 325 K. Above 295 K the density of free electrons would be sufficiently high to disrupt the condensate by decoherence and relaxation of the photoinduced polarons so that the state remains intact without any softening as its quantity diminishes to zero. However, *the condensate forms and is relatively stable at ambient temperature despite the large mass of a polaron in UO*_*2*_. The absence of any feature at the 30.8 K Neel temperature^69^,^70^ indicates the separation of the condensate from the host lattice. That the oscillation frequency is ~2 THz for all fluences and temperatures indicates that this mode is an intrinsic property of the condensate. If it represents or is associated with the lowest energy level of the state giving the condensate, THz relative to the 0.1 Hz frequencies typical of BECs would give a T_c_ of the order of 8000 K^62^. This is 27 times higher than the indicated value of T_c_ ~ 300 K found here, but sufficiently close relative to the factor of 10^8^–10^10^ difference with a conventional atomic condensate to be consistent with finding coherence through such a high temperature. This value is also consistent with its loss from competition with the scattering and other thermal processes.

Applying this same interpretation to our earlier report^22^, if the unusual peak in the transformed time domain spectrum from optically pumped and interrogated UO_2_ is assigned to an oscillation of a condensate instead of to a non-UO_2_ phonon, similar results are obtained. That experiment also found strong dependence on the excitation channel, with μs lifetimes for both 5*f* and 6*d* states but a gap-opening transition in an IGS only for the former. In addition, however, if the observed ~12 GHz signal is coupled to T_c_ as the lowest collective energy level of a condensate, its derived 45 K is almost exactly the 50–60 K of the transition that creates this new state. The high frequencies associated with excitations or transformations of the photoinduced UO_2_ condensates therefore appear to be directly correlated with their high temperatures.

*Microwave absorption in UO*_*2+x*_. Free charges and spins occur in UO_2+x_, perhaps even in stoichiometric UO_2.00_, via the low energy of the IGS and their dynamical attributes and involvement in the condensate. Their collective properties were elucidated by magnetic susceptibility and X band EPR spectroscopy, although in the context of condensates and other collective phenomena microwave spectroscopy in a magnetic field would be probing phenomena other than unpaired spins. Radiowave absorption has been used to identify and characterize the different states that occur in fermionic gases and condensates created by the application of a magnetic field^31^,^32^,^33^,^34^,^35^. The higher microwave energies are consistent with the higher temperatures and energies already noted in the pump-probe spectroscopies.

**Figure 4:**
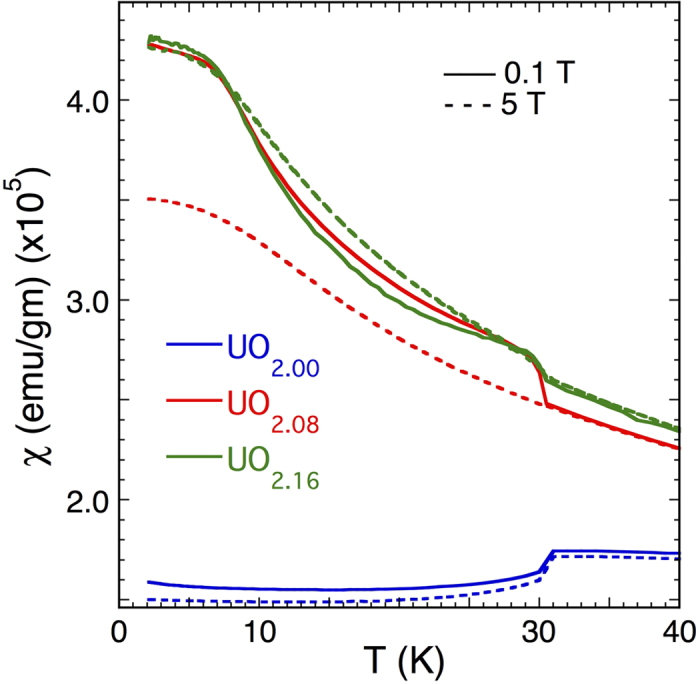
Magnetic susceptibility of UO_2+x_ from 2–40 K at 0.1 and 5 T. This temperature range shows the region of interest, no features occur at higher temperatures.

**Figure 5:**
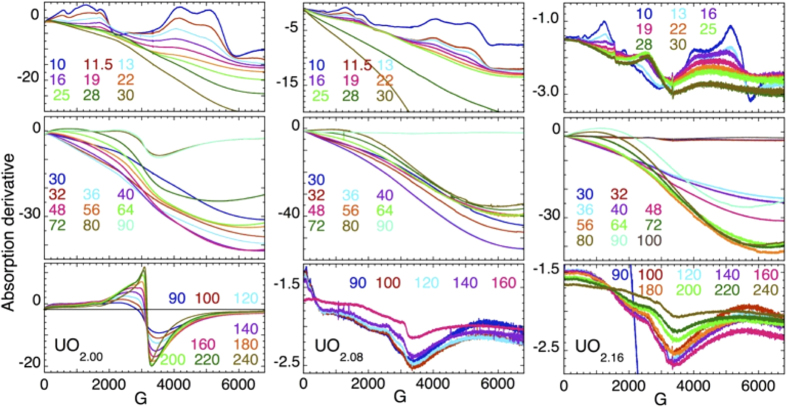
Microwave absorption spectra of UO_2.00_ (left), UO_2.08_ (center), and UO_2.16_ (right) at indicated temperatures. These are organized over temperature ranges where the spectra are sufficiently similar to be plotted on the same scale, concomitantly demonstrating the presence of different states associated with the different types of spectra.

X-ray diffraction patterns show the known phase separation between the UO_2_ and the slightly contracted U_4_O_9_ domains^71^,^72^, while EXAFS shows the phase separation and the multisite U-O distribution with the short U-O bond^73^ diagnostic for the oxo moiety. The magnetic susceptibility of UO_2.00_ (Fig. 4) matches previous results, with an antiferromagnetic ordering transition occurring at 30.8 K^74^,^75^. The 5*f*^2^ configuration of the non-Kramers U(IV) ion will give an EPR spectrum when the U site is distorted either statically to give a symmetric signal or dynamically to give an asymmetric one with widths around 100 G and g values up to 4.6 at 77 K^76^. After scrupulous cleaning of the cryostat we reproducibly measured the spectra shown here (Fig. 5) that exhibit much greater complexity than those from non-interacting U(IV) paramagnetic centers^77^,^78^. This complexity impedes integration of the signals that nevertheless represent only a fraction of the U. The UO_2.16_ spectra around 30 K resemble the small regions of those that were reported earlier^79^ for oxidized UO_2_ at 10 K. Some spectral features occur in all three samples while others vary with the U:O ratio. In contrast to only the two phases found by diffraction, susceptibility, and other bulk spin probes, the microwave absorbance is consistent with the EXAFS, another local probe, in showing a number of distinct states^77^,^79^.

The spectra that are possibly recognizable are those of UO_2.00_ at g = 2.1 for T ≥ 90 K. These exhibit a flat baseline and a signal that is similar in shape to that expected from a geometrically distorted S = 1/2 system. This could be from the individual polarons, whose increasing amplitude with temperature implies that they are forming from a lower energy EPR-silent state. Like most of the features in these spectra, its width is far too large for simple dipolar broadening^23^, and would therefore typically signify ferromagnetism. In the susceptibilities of UO_2.08_ and UO_2.16_ (Fig. 4) there appears to be a systematic decrease in effective moment and Weiss temperature with increasing x. The high temperature Curie-Weiss-like susceptibilities show monotonic decreases of the effective moments and Weiss temperatures with increasing x; viz., 3.25 μ_B_/U (257 K), 2.9 μ_B_/U (185 K), and 2.7 μ_B_/U (140 K) for x = 0, 0.08 and 0.16, respectively. The AFM transition occurs at the same temperature for all three compounds. However, the direction is inverted for the doped ones, although the magnitudes of the step are comparable and that of UO_2.16_ less than UO_2.08_. It is also suppressed at high fields, being almost eliminated for UO_2.08_. Since the susceptibility shows that these materials are not ferromagnetic in the normal sense the inversion in direction could signify a change in the canting angle of the U spin vector in the UO_2_ domains. The canting induced by addition of extra oxygen would be minimal, θ ≈ 5.6 × 10^–4^/1.74. The abrupt roll over of χ(T) at 6–7 K in lower fields could be another reorientation of the ordered moment. However, this type of flattening or rollover in the magnetization at low T following an abrupt rise was found in TiCuCl_3_, where it contributed to the interpretation of a magnon BEC in this system^4^. In the microwave spectrum of UO_2.16_ the dip near 3400 G is still present, reaching its greatest amplitude at 180 K similar to the UO_2.00_ spectra from 90–140 K. However, the rising portion at lower field of this particular signal is absent in the UO_2.16_ spectrum. This signal in the UO_2.08_ spectrum appears as a mixture of the two, reflecting the UO_2+δ_-U_4_O_9–δ_ phase separation as seen in the diffraction pattern. Two other very broad and complex signals are observed below 25 K in all three samples, albeit in somewhat modified form, at 0–2500 and 3400–5000 G.

**Figure 6:**
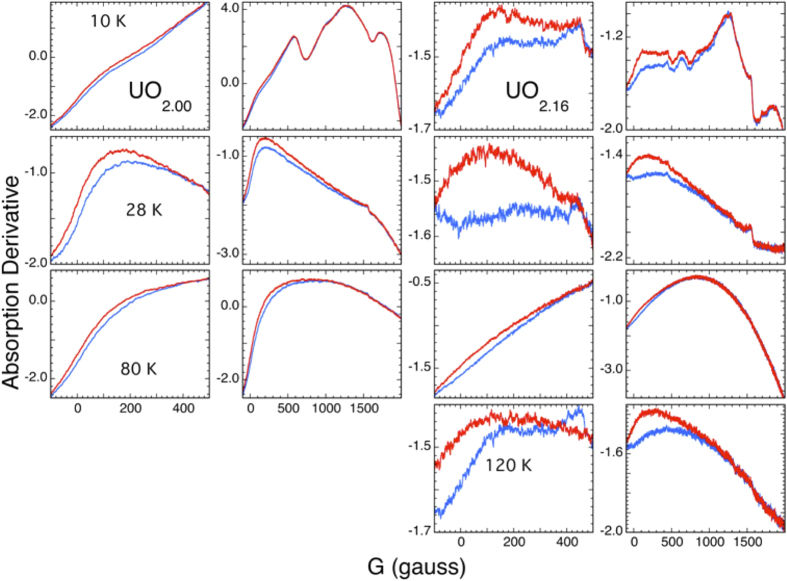
Microwave absorbance hysteresis results for UO_2.00_ (left two columns) and UO_2.16_ (right two columns) at 10, 28, 80, respectively, rows 1, 2, and 3, and 120 K for UO_2.16_. The plots are over the same range as the sweep. The blue traces are the upfield sweep and the red ones are downfield.

The distinctive condensate signature is the extended ramp in amplitude starting near zero field (Figs 5 and 6). This broad increase towards lower field is obscured in the spectra measured below 25–30 K because it is superimposed on the broad feature that is mostly absent by ~20 K. Comparing the baselines on the two sides of this feature shows, however, that the zero field background is larger so the ramp is still present under the peaks. This ramp becomes much larger through 50–60 K and diminishes again by 80–90 K where it is smaller in UO_2.08_ and UO_2.16_ (Fig. 5) and absent in UO_2.00_ with its flat baseline in this temperature range. This non-specific, low field microwave absorption has on occasion been observed in ferromagnets, but is also a signature of the superconducting phase in cuprates^80^. These two origins are differentiated by the direction and shape of the hysteresis when the field is reversed by scanning it through 0 G. For cuprates, a hysteresis curve generated by sweeping to lower field through and past the field reversal (G = 0) exhibits a diminished amplitude on the return when swept from negative to positive G and back, whereas ferromagnets show hysteresis in the opposite direction^24^. Since the temperature-field cycling path and doping of the material affect the microwave spectrum, the signal may not always drop through zero field or return to its origin, as found here (Fig. 6). In cuprates this microwave absorption originates in the transformation of the vortices from one pattern and type to another and are specific to the planar atom layers in those compounds^27^. Analogously, in the RF rather than microwave region, condensates give similar ramps to lower energy from interactions with defects^35^ and also most likely from vortex dynamics.

Following initial cooling of the UO_2+x_ samples to 80 K in zero field and then subsequent changes to 10, 28, and 120 K in field, when swept from −100 G through 100–6000 G and back all three samples (Fig. 6 shows results for UO_2.00_ and UO_2.16_) exhibit a difference in amplitude throughout the entire sweep that is even superimposed on other features – like cuprates – but with the return downfield sweep higher in amplitude than the upfield one – unlike cuprates. The collective microwave dissipation in UO_2+x_ is therefore of a novel type. The extension of the differential absorption to higher field as the spectral range is increased is a special and possibly unique property of cuprates where the flux line/vortex dis- and reordering from superconducting domains is continuous in energy. The opposite pattern, no change in the field where the hysteresis loop closes so that it is relatively symmetric around zero is characteristic of ferromagnets where the reversal of the magnetization occurs at a well defined energy^23^,^24^,^80^. Vortices and their associated lattices and subsequent dis- and reordering are, of course, endemic to all condensates^28^,^29^,^30^, not only superconductors. This includes magnon condensates^66^, which would corroborate the presence of a polaronic condensate whose unpaired atomic spins would be coupled in the composite bosons and that would give the very broad dissipation. That the direction of the hysteresis is that of ferromagnets and not cuprates is consistent with the field interacting directly with the particles as in fermionic condensate formation instead of with excluded/expelled flux lines. These UO_2+x_ spectra are also notable in not exhibiting any features coincident with the AFM ordering transition. It is therefore unsurprising that these spins or other phenomena that give the spectra are also decoupled from those giving the anisotropy in the thermal conductivity^81^. The absorbing species are separated from the UO_2.00_ domains despite the UO_2.00_ spins being affected by their presence. A notable feature of this separation, however, is that the UO_2.00_ spin system and the condensate domains are presumably intermingled within the crystal on the scale of the domain sizes. In addition, the many features of the spectra and their complicated temperature dependence are analogous to the RF absorbance of the ultracold fermionic systems, where the different states including those from defects give very similar kinds of behaviors^31^,^32^,^33^,^34^,^35^,^82^. The totality of these spectra, with the possible exception of the high temperature UO_2.00_ signal, may therefore originate in precisely the type of phenomena observed in fermionic condensates.

### Implications

These results validate the conjecture, based on the observed superfluid atom tunneling and broadening of the electronic states in UO_2+x_ and the extremely long lifetimes of the collective phases of the excited states and their coherent oscillations in photodoped material^22^, that UO_2(+x)_ hosts polaronic condensates. The condensate emerges from individual or aggregated quasiparticles whose collective interactions are enabled by dynamically expanded wavefunctions extending into the UO_2_ host. This interpretation of the unique oscillation observed in the IGS pump-TTDS probe experiment is corroborated by the presence of distinct mesoscale domains of a coherent quasiparticle fluid, as derived from our prior structural measurements and observed for other quasiparticle condensates^5^,^63^.

**Figure 7:**
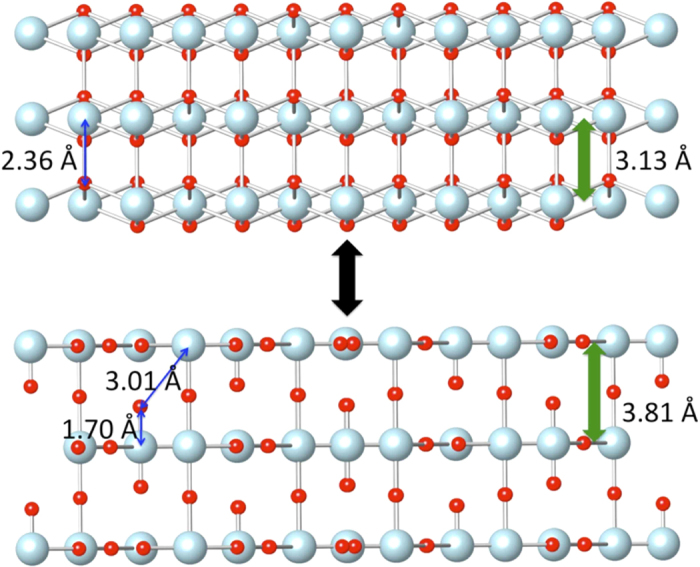
The collective dynamical polaron in UO_2+x_. Within a UO_2_-based U-(111) phonon, the “neutron-type” U_4_O_9_ structure with U(V) sites is favored when the dynamical interplane spacing is contracted (upper), whereas the “x-ray-type” excited state U_3_O_8_-like structure containing U(VI) formed via the 2 U(V)↔U(IV)+U(VI) disproportionation reaction, with the U(IV) transported to UO_2_-like domains, is stabilized on the expanded side of the vibrational excursion (lower). This schematic captures, e.g, the superfluidity of the O^2–^ ion motions but does not accurately model the disorder in the U-U distribution that would be necessary to allow the spacing to become less than that in UO_2_ so as to conserve the overall density.

A remarkable characteristic of these polaronic condensates is their concomitant high mass and temperature, up to 300 K, indicating a novel coherence mechanism. Insight into this may be found in the structural measurements that demonstrate that the dynamics resides in a disproportionation reaction, 2 U(V)↔U(IV)+U(VI). This can be combined with the finding that stress relief in UO_2_ occurs in its [111] plane^83^,^84^. The coherence would result from the coupling of this or an alternative boson-forming interaction to the motion of a UO_2_ (111)-type acoustic phonon in which the cubic U(IV/V) “neutron” configuration is more stable when the [111] U planes are on the compressed and the layered U(IV/VI) “x-ray” conformation on the expanded sides, respectively, of the vibrational motion (Fig. 7). Concerted motion is then imposed by the geometric and elastic constraints of the crystal that cause the energy to decrease as the number of atoms involved increases and as their motions become synchronized because of reduction of the strain energy from the layered domains within the cubic host. This process involving a non-degenerate double well potential could be considered as a solid state analog of a Feshbach resonance. The fluorite structure with unpaired U(V) electrons is the open channel, connected to the layered structure that is the closed U(IV–VI) channel via this phonon. When excited, not only do the energies correspond but also the Franck-Condon barrier is eliminated when the extended displacement of the atoms intersects with the ground state of the higher energy structure. The system thus resonantly interconverts the kinetic energy of the excited, fermionic vibrational state into the enthalpy of the excited bosonic structural state, oscillating between these two states in coincidence with the phonon controlling the interplanar separation that drives the transformation between the fluorite and layered structures. The role of Feshbach resonances in the formation of the bosonic molecules that compose fermionic condensates has been extensively studied^31^,^32^,^37^,^64^,^65^,^82^,^85^,^86^,^87^,^88^, concluding that “…near the Feshbach resonance, theory predicts coherent oscillations between the…states^85^…” Insofar as the theories developed for ultracold Fermionic liquids and condensates are applicable to solid state phenomena^16^,^17^,^18^,^21^,^89^, the coherence would therefore be an expected outcome of this chemical reaction that was observed directly in our structural measurements. This supports the idea that the requirement for Bose-Einstein systems that all of the constituent particles be in the same state may be achieved in multiple ways, with the use of ultralow temperatures to place them in the ground state being a matter of convenience rather than necessity.

The coherence and subsequent condensate have additional consequences. One is the superfluidity of the atoms across the oscillation that allows the requisite rapid rearrangement of the O atoms without a barrier. A second is the stability of the condensate that appears to cause the formation of O-enriched domains on irradiation^84^,^90^ and even, as demonstrated here, spontaneously in undoped UO_2.00_ via metal-to-metal charge transfer reactions to produce the required U(V). Another is the formation of the vortices and other states of the system^91^ that account for the microwave dissipation. UO_2+x_ is one more example of the valence-structure-coupled polaron dynamics, but an extreme one whose non-adiabatic spin-charge-lattice fluctuations synchronize to become coherent oscillations of the condensate. The persistence of the condensate to high temperature would therefore occur because this reaction in UO_2(+x)_ is a non-degenerate one to an excited state originating in unique aspects of the structural chemistry of U^73^ (and Pu^92^) oxides. This would be in contrast to the dynamical polarons in other mixed valence oxides that involve charge transfer between identical ground state structures. That a more limited version of these behaviors is exhibited by cuprates^89^, with possible hints in related materials^38^, supports the idea that a related mechanism might be active in those systems as described in the introduction. Another pertinent finding is a recent report of mixed BCS-BEC superconductivity in FeTe_0.6_Se_0.4_^93^ involving low lying IGS^15^ that would corroborate two-state theories^16^,^19^,^20^. Although for UO_2(+x)_ the BEC is composed of electrons and not atoms, it is a demonstration that BECs are extensible to the fundamental components of condensed matter^21^,^89^. We also call attention to the anomalous broadening of the spectral features in the O XAS of UO_2+x_ relative to UO_2.00_^22^. A corresponding enhancement of the dispersion in the now partly filled U 5*f* band that would be at the Fermi level would produce much more complicated Fermi surface topology than the relatively flat band in UO_2.00_ before doping and also contribute to forming and stabilizing a condensate via exchange. These results supporting BEC-formation in UO_2(+x)_ are the next step on this path. To the extent that the proposed mechanism is correct, we note that the Feshbach resonance occurs in the crossover or pseudogap regime, which would suggest that the UO_2(+x)_ condensate could be tuned in both the BEC and BCS directions.

## Methods

*UO*_*2+x.*_ The UO_2_ crystal for the TTDS measurements was the same as used earlier and the UO_2+x_ powder samples were prepared identically^22^.

### Terahertz Time Domain Spectroscopy

In our experiment, we investigated 200 μm thick highly polished uranium dioxide samples. The sample was fixed in a rotational mount to allow the sample orientation to be varied with respect to the laser polarization. A 6 mJ, 40 fs laser pulse was first split with a 90/10 beamsplitter. The stronger portion of the beam was sent through a half-wave plate and then focused with a 12.5 cm focal length lens into an argon filled gas cell for the broadband THz pulse generation .The cell was kept at a pressure of ~700 Torr which was chosen to maximize the THz pulse energy and bandwidth. Also located inside the gas cell was a barium borate (BBO) crystal. This was placed ~2 centimeters before the laser focus to avoid damage to the crystal while also producing a sufficient amount of second harmonic for the THz pulse production. The half-wave plate and the BBO orientation were chosen to provide the maximum THz pulse energy along with a linear p-polarized pulse after the gas cell. Typically we estimated the THz pulse energy at nearly 1 μJ. This was measured by directing the THz photons onto a pyroelectric detector and calibrating the response with a helium-neon laser pulse of known energy. The end of the gas cell contained a silicon filter to remove the residual 800 nm and 400 nm laser light while passing as much of the THz radiation as possible. From there, the THz pulse passed through an 8-f optical arrangement of 2 inch, gold-coated, parabolic mirrors. The UO_2_ sample was located within this arrangement, but was removed when the original THz pulse was to be measured. The THz pulse was finally focused between our detection slits. Meanwhile, the remaining portion of the original laser pulse passed through a 50/50 beamsplitter. The transmitted half of the beam passed through a delay stage, was rotated to p-polarization, and was eventually focused between the detection slits and combined with the THz pulse. The total pulse energy of this beam could be as high as 300 μJ although this was usually further attenuated with a variable ND filter. The slits had a 500 Hz, 2 kV bias applied and it was to this signal that a lock-in amplifier was locked to. A weak second harmonic field was generated between the slits and modulated by the mixing of the two beams. The second harmonic signal then passed through a 400 nm bandpass filter and was detected with a photo multiplier tube. Together, the THz and 800 nm laser beams allowed a time-domain measurement of the THz waveform with the air biased coherent detection (ABCD) method.

The remaining portion reflected from the 50/50 beamsplitter also passed through a delay stage and was focused onto the UO_2_ sample. This served as the pump pulse for our experiments. Within this portion of the beam path was also located a half-wave plate, variable ND filter, and another BBO crystal for possible 3.1 eV pumping of the UO_2_ sample in the future. Additionally, a reflection is seen at ~6 ps resulting from a Fabry-Perot effect from the sample itself. All subsequent data was truncated in the time domain before the reflection to avoid artificial spectral modulations after converting the time-domain signal to the frequency domain with an FFT. Reducing the time window to ~5 ps after time zero reduced the low frequency resolution below 1 THz which was inconsequential to our data accumulation. The pump beam was not collinear with the THz probe beam. The sample was pumped at an angle that could not be captured by the collection optics for the THz, preventing the signal from originating in THz emission stimulated by the pump beam.

### Phonon calculations

Phonon calculations were performed for UO_2_ through the direct frozen-phonon method using the Vienna Ab-initio Simulation Package (VASP) combined with the phonopy package on supercells consisting of 4 × 4 × 4 primitive cells (192 atoms). To obtain a good description of the electronic structure of this highly correlated system, we have used density functional theory including an additional Hubbard term (DFT+U). The gradient generalized approximation (GGA) was chosen for the DFT exchange-correlation functional. In the employed GGA + U approach, the Hubbard and exchange parameters, U and J, respectively, are introduced to account for the on-site Coulomb correlations between the uranium 5f electrons; the +U term also helps to remove the self-interaction error. We have chosen a Hubbard U value of 4.5 eV and an exchange parameter J value of 0.54 eV, which have been shown to provide good results. To deal with the problem of occurring metastable states when using the DFT+U method, we have used the occupation matrix control (OMC) technique proposed by Dorado *et al.*^94^ The electron ionic-core interaction on the valence electrons in the systems has been represented by the projector augmented wave potentials (PAWs). For U and O atoms, the (6*s*, 7s, 6p, 6d, 5f) and (2*s*, 2*p*) states were treated as valence states. A plane-wave basis with an energy cutoff of 510 eV was used to expand the electronic wave functions. The Brillouin zone integrations were performed on a special k-point mesh generated by the Monkhorst Pack scheme (6 × 6 × 6 in the bulk and 2 × 2 × 2 for the phonon supercells), and a Gaussian smearing of σ = 0.1 eV was used. The electronic minimization algorithm used for static total-energy calculations was a blocked Davidson algorithm.

In the phonon calculations we corrected the long range macroscopic electric field generated by collective ionic motions near the gamma point by adding a non-analytical term to the dynamical matrix.

### Magnetic susceptibility

Magnetic measurements were performed in a Quantum Design SQUID magnetometer over the temperature range 2 ≤ T ≤ 350 K and in various fixed magnetic fields between 1 and 50 kOe. For these measurements, a powder of UO_2+x_ was sandwiched between two plastic discs whose susceptibility was determined independently and subtracted from the magnetic response of the sample plus discs. In plots shown, χ is defined as the magnetic moment of the sample divided by applied field, M/H.

### Electron Paramagnetic Resonance (EPR) Spectroscopy

All samples for EPR spectroscopy were prepared in an inert nitrogen atmosphere glove box equipped with a liquid nitrogen fill port to enable sample freezing to 77 K within the glove box. EPR samples were prepared in 4 mM OD thin wall precision Suprasil quartz screw cap EPR tubes from Wilmad Labglass. All samples for EPR spectroscopy were prepared in the solid state. X-band EPR spectra were recorded on a Bruker EMXplus spectrometer equipped with a 4119HS cavity and an Oxford ESR-900 helium flow cryostat. The instrument parameters employed for all samples were as follows: 2 mW power; time constant 41 ms; modulation amplitude 12 G; 9.38 GHz (10K spectra)/9.83 GHz (298K spectra); modulation frequency 100 kHz.

## Additional Information

**How to cite this article**: Conradson, S. D. *et al.* Possible Demonstration of a Polaronic Bose-Einstein(-Mott) Condensate in UO_2(+x)_ by Ultrafast THz Spectroscopy and Microwave Dissipation. *Sci. Rep.* **5**, 15278; doi: 10.1038/srep15278 (2015).

## Supplementary Material

Supplementary Information
