# Supplementary material for: Possible Demonstration of a Polaronic Bose-Einstein(-Mott) Condensate in UO2(+x) by Ultrafast THz Spectroscopy and Microwave Dissipation
Source: Sci Rep. 2015 Oct 16;5:15278. doi: 10.1038/srep15278 (PMC4607891; doi:10.1038/srep15278)
Supplement: Supplementary Information [file srep15278-s1.pdf]

Possible Demonstration of a Polaronic Bose-Einstein(-Mott) Condensate in $UO_{2(+x)}$ by Ultrafast THz Spectroscopy and Microwave Dissipation

Steven D. Conradson, Steven M. Gilbertson, Stephanie L. Daifuku, Jeffrey A. Kehl, Tomasz Durakiewicz, David A. Andersson, Alan R. Bishop, Darrin D. Byler, Pablo Maldonado, Peter Oppeneer, James A. Valdez, Michael L. Neidig, George Rodriguez

Supplementary Information

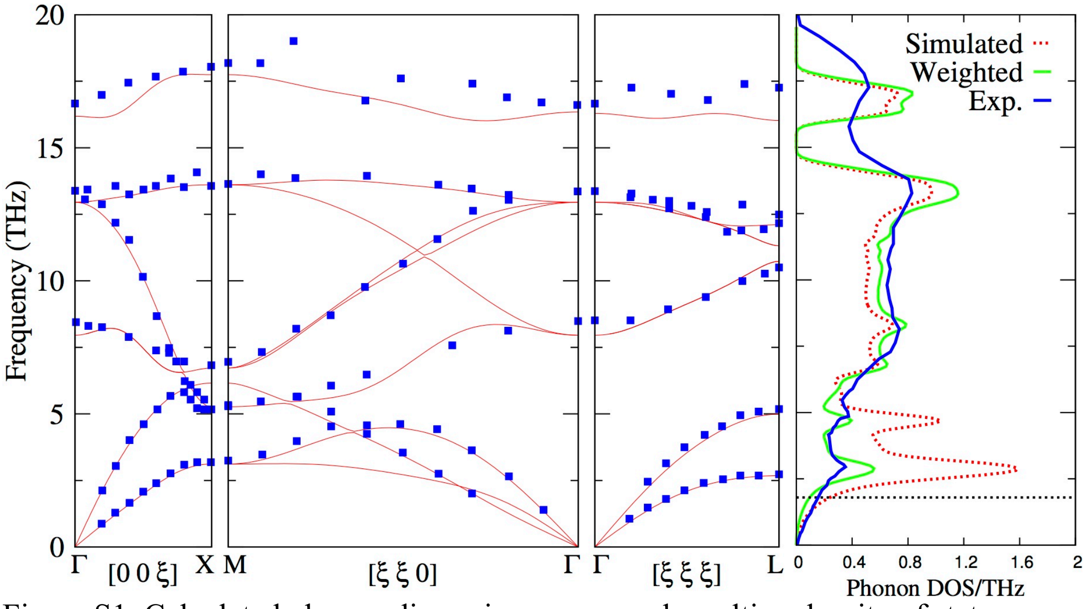

Figure S1. Calculated phonon dispersion curves and resulting density of states.
